# Comparison of Functional Outcome Between Intra-Articular Injection of Corticosteroid Versus Platelet-Rich Plasma in Frozen Shoulder: A Randomized Controlled Trial

**DOI:** 10.7759/cureus.20560

**Published:** 2021-12-21

**Authors:** Hafiz Faisal Shahzad, Muhammad Taqi, Syed Faraz Ul Hassan Shah Gillani, Faisal Masood, Munawar Ali

**Affiliations:** 1 Orthopedic Unit-1, Mayo Hospital, Lahore, PAK; 2 Orthopedics and Traumatology, Mayo Hospital, Lahore, PAK; 3 Orthopedic Surgery, Mayo Hospital, Lahore, PAK; 4 U.S. Department of Veterans Affairs, University of Oklahoma Health Sciences Center, Oklahoma, USA

**Keywords:** intra-articular platelet-rich plasma, vas score, prp, intra-articular steroids, frozen shoulder

## Abstract

Background

In this study, we compared the functional outcome of intra-articular injection of corticosteroid versus platelet-rich plasma (PRP) in patients with frozen shoulder (FS).

Methodology

This randomized controlled trial was conducted in the Department of Orthopedics, Mayo Hospital, Lahore, from January 2018 to December 2018.A total of 202 patients with FS aged 40 to 70 years were included. Patients with medical comorbidities such as chronic liver disease (assessed on history and serum bilirubin >2.0 mg/dl), chronic renal failure (assessed on history and serum creatinine >1.5 mg/dL), and chronic steroid use were excluded. Employing an anterior approach, subjects in groups A and B received one intra-articular injection of 2 mL PRP and 2 mL (80 mg) methylprednisolone acetate, respectively. Age, gender, duration of disease, and pre-injection and post-injection range of motion (ROM) (flexion, extension, abduction, external rotation, and internal rotation) were assessed. The University of California at Los Angeles Shoulder Score (UCLA) and visual analog scale (VAS) scores were measured and compared before and after the injection. All patients were followed at regular intervals post-therapy and the final functional outcome was measured after 12 weeks of therapy.

Results

Data were analyzed using SPSS version 20 (IBM Corp., Armonk, NY, USA). A p-value of 0.05 was considered significant when comparing flexion, abduction, external rotation, and internal rotation in both groups using the independent t-test. The ROM in group A (intra-articular PRP) improved significantly (p < 0.05) after injection compared to group B (intra-articular corticosteroid). The ROM after PRP for abduction was 147.09 ± 7.78, forward flexion 154.52 ± 6.48, external rotation 71.59 ± 7.43, and internal rotation 59.20 ± 3.96. The ROM in the steroid injection group for abduction was 129.07 ± 4.72, forward flexion 127.14 ± 7.87, external rotation 56.27 ± 5.93, and internal rotation 48.86 ± 4.90.

Conclusions

Intra-articular injection of PRP resulted in a substantial improvement in the VAS score, UCLA, and ROM when compared to intra-articular corticosteroid injection in patients with FS.

## Introduction

Frozen shoulder (FS) is a common disease with high morbidity. Codman was the first to coin the phrase “frozen shoulder” in 1934 [1]. Because of adhesion and fibrosis in the glenohumeral (GH) capsule, which reduces joint space, FS affects the GH joint and restricts active and passive mobility. FS is estimated to have a 3-5% incidence rate and a 10-20% frequency among adults [2]. According to a previous study, there is no difference in pain and impairment between FS patients with and without diabetes [3]. Although this condition has a benign stage and recovers after two or three years, in certain circumstances, the symptoms and signs of the disease remain permanent. After three years, up to 40% of patients, to our knowledge, have persistent problems [4].

Several alternative therapies have been suggested such as benign neglect, oral corticosteroids, intra-articular injections of corticosteroids, intra-articular injections of hyaluronic acid, physical therapy, deep heat regimens, manipulation under anesthesia, hydro-dilation, and arthroscopic release; however, the best treatment option remains debatable [5]. One of the most widely utilized treatments for FS is intra-articular corticosteroid injection [6,7].

As there is a paucity of literature available to compare functional outcomes of steroids with platelet-rich plasma (PRP), the goal of this study is to establish facts regarding the benefits and possible complications of intra-articular corticosteroid injections versus PRP injections. This study will help in evaluating and devising the better treatment option among the two approaches to ensure that these patients return to their routine life as soon as possible.

## Materials and methods

This randomized controlled trial was conducted in the Department of Orthopedic Surgery and Traumatology employing a probability simple random selection approach. Randomization was done using the lottery method and a transparent envelope was prepared; however, it was blinded by the chief researcher. Ethical approval was obtained from the university. The sample size was calculated to be 202 including 102 cases in group A and 100 cases in group B, with a 5% level of significance and 95% power. Patients between the ages of 40 and 70 years with a ≤6-month history of FS and loss of 100° ROM were included. Patients with a history of prior operations of the shoulder joint, medical comorbidities such as chronic liver disease (serum bilirubin >2.0 mg/dL), chronic renal failure (serum creatinine >1.5 mg/dL), chronic steroid use, old healed fractures, and drug allergies to steroids were excluded.

After obtaining approval from the institutional review board, informed written consent was obtained and the study goal was explained. Randomization was done using the lottery method, and patients were randomized into two groups A and B. All selected individuals were given the option of selecting a slip from mixed slips (half of the slips containing the letter “A” and the other half containing the letter “B”) and were assigned to the selected group. Using the anterior approach, subjects in groups A and B received one intra-articular injection of 2 mL PRP and 2 mL (80 mg) methylprednisolone acetate, respectively, without ultrasound guidance. Throughout the procedure, aseptic conditions were maintained. Centrifugation was used to separate PRP from autologous blood. After the injection, the participants were sent home with instructions to limit shoulder joint movement for at least 48 hours and use paracetamol as a pain reliever. During each injection, adverse effects were reported. ROM was measured using a goniometer in anteroposterior and lateral planes. Patients were followed up in the outpatient department at four, eight, and twelve weeks post-injection. After two days, shoulder exercises were started. In both groups, the injection was administered by the researcher himself. All patients were followed at regular intervals post-therapy, and the final functional outcome using clinical examination was measured after 12 weeks of therapy. The improved ROM of ≥30° was considered clinically significant.

The means and standard deviations for age, illness duration, and pre- and post-injection ROM (flexion, abduction, external rotation, and internal rotation) were calculated. Gender and diabetes mellitus (yes/no) were used to determine frequency and percentages. In both groups, an independent t-test was performed to compare flexion, abduction, external rotation, and internal rotation, and a p-value of 0.05 was considered statistically significant. The University of California at Los Angeles Shoulder Score (UCLA) and visual analog scale (VAS) scores were measured and compared before and after the injection. Through stratification, effect modifiers such as age, gender, illness duration, and diabetes mellitus (yes/no) were controlled, and the post-stratification independent t-test was used. P-values of less than 0.05 were considered significant.

## Results

In group A, 41.38% of the participants were males and 56.82% were females, and in group B, 40.91% of the participants were males and 59.09% were females. The male-to-female ratio was 1:1.4. The mean age of the patients in group A was 70.41 ± 4.67 years and in group B was 57.0 ± 7.74 years. The age range in this study was 40 to 70 years, with a mean age of 57.24 ± 7.71 years in both groups (Table 1). Of the 202 patients, the mean duration of disease was 5.15 ± 1.49 months (Table 2).

**Table 1:** Demographic characteristics of the study participants. SD: standard deviation

Variables	Mean age SD (years)			Duration of disease			Gender	
	40–55	56–70	Mean	<5 months	>5 months	Mean	Male	Female
	Number of patients			Number of patients			Percentage of patients	
Group A (102)	47	55	52.41 ± 2.67	49	53	5.57 ± 1.49	41.38	56.82
Group B (100)	55	50	53.0 ± 3.74	68	32	4.86 ± 1.50	40.91	59.09

**Table 2:** Comparison between pre-injection ROM and post-injection ROM. ROM: range of motion; SD: standard deviation

ROM	Pre-injection ROM				P-value	Post-injection ROM				P-value
	Group A (102)		Group B (100)			Group A (102)		Group B (100)		
	Mean	SD	Mean	SD		Mean	SD	Mean	SD	
Forward flexion	83.41	22.34	83.74	21.49	0.925	154.52	6.48	127.14	7.87	0.0001
Abduction	65.13	17.61	64.98	17.18	0.957	147.09	7.78	129.07	4.72	0.0001
External rotation	38.33	12.19	38.02	11.79	0.904	71.59	7.43	56.27	5.93	0.0001
Internal rotation	28.33	22.19	28.02	21.79	0.947	59.20	3.96	48.86	4.90	0.0001

In group A, pre-injection mean ROM was forward flexion (83.41°), abduction (65.13°), external rotation (38.33°), and internal rotation (28.33°). In group B, pre-injection mean ROM was forward flexion (83.74°), abduction (64.98°), external rotation (38.02°), and internal rotation (28.02°). There was no significant difference between the two groups concerning the pre-injection ROM (forward flexion, abduction, external rotation, and internal rotation). As shown in Table 3, group A (intra-articular PRP) had a significant improvement (p = 0.05) in ROM compared to group B (intra-articular corticosteroid). In the study, the ROM after PRP (group A) for abduction was 147.09 ± 7.78, forward flexion 154.52 ± 6.48, external rotation 71.59 ± 7.43, internal rotation 59.20 ± 3.96. In the steroid injection group (group B), the ROM for abduction was 129.07 ± 4.72, forward flexion 127.14 ± 7.87, external rotation 56.27 ± 5.93, and internal rotation 48.86 ± 4.90. The acquired ROM in diabetics and non-diabetics is presented in Table 4. The pre-treatment VAS scores and UCLA were statistically similar in both groups. In all groups, there was a substantial improvement in pain and shoulder function after therapy (Table 5). The VAS score of group A after therapy was considerably lower than that of group B. The UCLA of group A was substantially higher than that of group B (p = 0.002 and p = 0.004, respectively) after therapy.

**Table 3:** Stratification according to the duration of disease. ROM: range of motion; SD: standard deviation

ROM	Pre-injection <5 months				P-value	Post-injection >5 months				P-value
	Group A (102)		Group B (100)			Group A (102)		Group B (100)		
	Mean	SD	Mean	SD		Mean	SD	Mean	SD	
Forward flexion	54.24	6.80	67.20	7.58	<0.001	154.78	6.31	127.0	8.75	<0.001
Abduction	47.29	6.99	88.03	5.07	<0.001	146.91	8.60	131.29	2.92	<0.001
External rotation	71.00	7.52	56.07	5.34	<0.001	72.13	7.46	56.71	7.25	<0.001
Internal rotation	59.10	4.30	48.93	4.96	<0.001	59.30	3.72	48.71	4.95	<0.001

**Table 4:** Comparison of ROM between diabetic and non-diabetic patients. ROM: range of motion; SD: standard deviation

ROM	Diabetic patients				P-value	Non-diabetic patients				P-value
	Group A (100)		Group B (100)			Group A (100)		Group B (100)		
	Mean	SD	Mean	SD		Mean	SD	Mean	SD	
Forward flexion	53.77	7.51	67.00	7.41	0.0001	154.84	6.11	127.23	8.31	0.0001
Abduction	48.46	7.88	78.00	3.96	0.0001	146.52	7.80	129.81	5.12	0.0001
External rotation	71.69	7.19	56.06	5.43	0.0001	71.55	7.64	56.42	6.36	0.0001
Internal rotation	60.15	3.60	50.00	5.02	0.0001	58.81	4.09	64.08	4.76	0.0001

**Table 5:** Comparison of shoulder pain and functions. VAS: visual analog scale; UCLA: University of California at Los Angeles Shoulder Score

Score	Pre-injection			Post-injection		
	Group A	Group B	P-value	Group A	Group B	P-value
VAS	8.9 ± 1.01	9.5 ± 0.6	0.06	0.85 ± 0.52	2.3 ± 1.6	0.004
UCLA	11.6 ± 1.64	11.9 ± 2.04	0.93	27 ± 1.9	23 ± 6.62	0.002

## Discussion

PRP is a small volume of plasma containing an autogenous concentration of human platelets. PRP has the ability to generate collagen and growth factors, as well as potentially boost stem cells, enhancing the healing process by providing large concentrations of alpha-granules carrying physiologically active subunits (such as vascular endothelial growth factor and transforming growth factor-β) to the areas of soft tissue damage [8,9]. Adhesive capsulitis is more frequent in the fifth and sixth decades of life, and people under the age of 40 should be evaluated for other medical conditions. There is no evidence of racial prejudice in the literature [10,11]. In our study, 37 (36.54%) males and 51 (63.46%) females made up a male-to-female ratio of 1:1.4. These findings corroborate the findings of several earlier studies, which reported that the incidence of FS is two times higher in females than in males [12,13]. Diabetes mellitus has been identified as one of the risk factors for FS in several previous studies, and its incidence has been reported to be significant in diabetic individuals [2].

When comparing group A (intra-articular PRP) to group B (control), the results showed that group A had a significant improvement (p = 0.05) in the ROM after injection (intra-articular injection of corticosteroid). In this study, the ROM after PRP was abduction 147.09 ± 7.78, forward flexion 154.52 ± 6.48, external rotation 71.59 ± 7.43, and internal rotation 59.20 ± 3.96 in comparison to abduction 129.07 ± 4.72, forward flexion 127.14 ± 7.87, external rotation 56.27 ± 5.93, and internal rotation 48.86 ± 4.90 in the steroid injection group. A study about the functional outcome of FS by Kothari et al. [11] showed that the active ROM after PRP was abduction 142.3 ± 22.9, forward flexion 145.5 ± 13.5, external rotation 80.2 ± 13.8, and internal rotation 57.5 ± 10.7 compared to abduction 129.7 ± 21.8, forward flexion 133.1 ± 18.5, external rotation 71.4 ± 18.3, and internal rotation 50.2 ± 13.4 in the steroid injection group [14]. The result of this study cannot be generalized because of its small sample size.

Rawat et al. investigated 32 patients with FS who received intra-articular steroid injections. After 12 weeks of follow-up, pain alleviation was statistically significant [15]. According to Shah and Lewis, multiple corticosteroid injections were helpful until 16 weeks among FS patients [16]. The required duration of treatment is greater compared to PRP injections. Aslani et al. [2] found that two consecutive treatments of PRP with a four-week gap increased the functional ROM and alleviated pain in a volunteer with FS. With PRP therapy, he saw a two-fold improvement in ROMs [17].

PRP and procaine were found to be helpful in treating FS in the study by Lin. The PRP treatment was more effective and lasted longer than the procaine treatment. The UCLA improved linearly in the PRP group but reduced to a lower level at the last three-month follow-up visit in the procaine group. Following one week, one month, and three months after the initial injection, VAS scores in both the PRP and control groups decreased [18,19].

This study had several limitations. Patients in various stages of FS were included in the study without stratification. The use of ultrasound for intra-articular injection was not evaluated. Moreover, the sample size was small. Multicenter controlled trials are required for more accurate results.

## Conclusions

In comparison to intra-articular corticosteroid injection, there is a substantial improvement in VAS score, UCLA, and ROM in patients with FS following intra-articular injection of PRP. Hence, we believe that intra-articular injections of PRP should be utilized frequently in the treatment of FS to achieve better outcomes.
